# Supplementary material for: Identification of the Xyloglucan Endotransglycosylase/Hydrolase (XTH) Gene Family Members Expressed in Boehmeria nivea in Response to Cadmium Stress
Source: Int J Mol Sci. 2022 Dec 17;23(24):16104. doi: 10.3390/ijms232416104 (PMC9785722; doi:10.3390/ijms232416104)
Supplement: Supplementary file 1 [file ijms-23-16104-s001.zip › ijms-2090158-supplementary.pdf]

Supplementary information

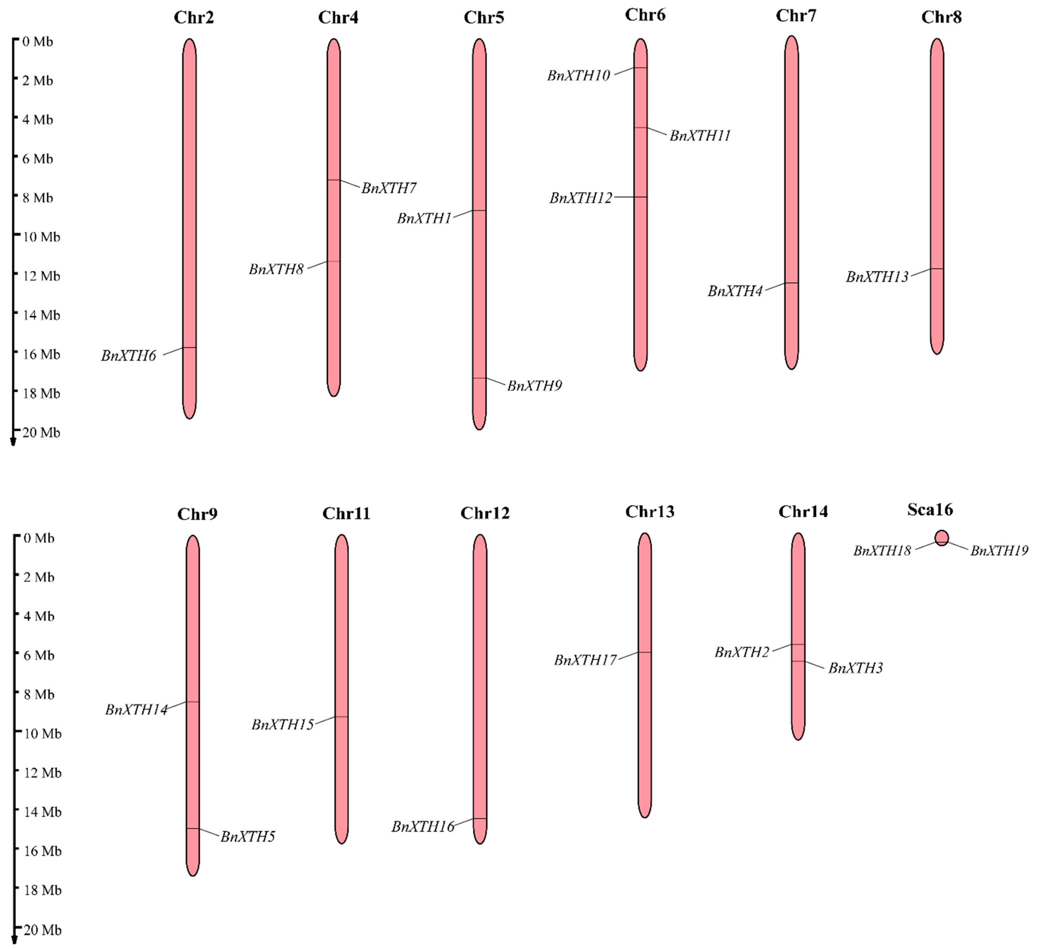

Figure S1. Chromosomal locations of *BnXTH* gene family members

Table S1. Sequence and SeqLogo of the Motif 1-10

Motif ID	Amino acid sequence encode	SeqLogo
Motif_1	FDPTKDFHTYSILWNPQRIIFLVDBVPIRVYKN	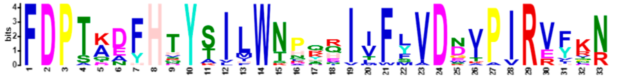
Motif_2	HDEIDFEFLGNRSGEPYTLQTNVYAQGKGDREQR	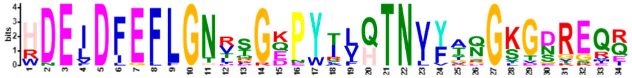
Motif_3	WATRGGLVKTDWSKAPFVASYRBFDADGCVV	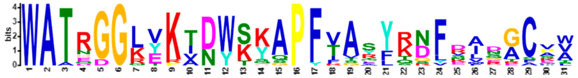
Motif_4	YLFGRFSMQIKLVPGNSAGTVTAFYLSSQ	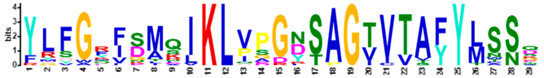
Motif_5	ZRRRLRWVRRKYMIYBYCTDTKRFPQGP	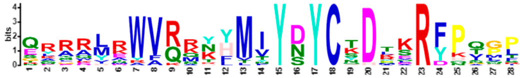
Motif_6	PKSQPMRIYSSJWBA	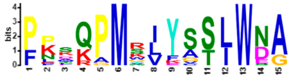
Motif_7	DGELLTLSLDKTSGSGFQSKN	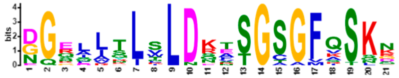
Motif_8	AGNFYKDFDITWGDDHAK	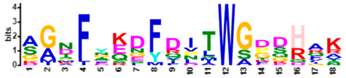
Motif_9	NRASNPANWWEGPAYQDL	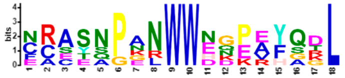
Motif_10	EAKGIPY	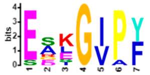

Table S2. Collinear genes among different species

Boehmeria nivea—Oryza sativa	*Boehmeria nivea—Sorghum bicolor*	*Boehmeria nivea—Arabidopsis thaliana*	*Boehmeria nivea—Populus trichocarpa*
BnXTH4-OsXTH30	*BnXTH1-SbXTH8*	*BnXTH6-AtXTH32*	*BnXTH6-PtXTH32*
BnXTH4-OsXTH29	*BnXTH14-SbXTH27*	*BnXTH6-AtXTH31*	*BnXTH6-PtXTH31*
BnXTH14-OsXTH27		*BnXTH7-AtXTH9*	*BnXTH8-PtXTH15*
		BnXTH1-AtXTH8	*BnXTH8-PtXTH2*
		BnXTH12-AtXTH3	*BnXTH7-PtXTH9*
		BnXTH12-AtXTH1	*BnXTH9-PtXTH15*
		BnXTH4-AtXTH30	*BnXTH1-PtXTH8*
		BnXTH4-AtXTH29	*BnXTH9-PtXTH2*
		BnXTH13-AtXTH26	*BnXTH9-PtXTH2*
		BnXTH5-AtXTH33	*BnXTH12-PtXTH2*
		BnXTH14-AtXTH28	*BnXTH11-PtXTH10*
		BnXTH14-AtXTH27	*BnXTH4-PtXTH30*
		BnXTH15-AtXTH16	*BnXTH4-PtXTH30*
		BnXTH15-AtXTH15	*BnXTH14-PtXTH28*
		BnXTH16-AtXTH32	*BnXTH14-PtXTH28*
		BnXTH17-AtXTH18	*BnXTH15-PtXTH2*
		BnXTH2-AtXTH7	*BnXTH15-PtXTH2*
		BnXTH2-AtXTH6	*BnXTH16-PtXTH32*
			BnXTH16-PtXTH31
			BnXTH16-PtXTH32
			BnXTH2-PtXTH6

Table S3. Cis-acting elements on the promoters of *BnXTHs*

Gene name	Element name	Function	**Group**
BnXTH1	ERE		Phytohormione reponsive
BnXTH1	Box 4	part of a conserved DNA module involved in light responsiveness	Growth and development
BnXTH1	Box 4	part of a conserved DNA module involved in light responsiveness	Growth and development
BnXTH1	Box 4	part of a conserved DNA module involved in light responsiveness	Growth and development
BnXTH1	WUN-motif	wound-responsive element	Abiotic and biotic stress
BnXTH1	Box 4	part of a conserved DNA module involved in light responsiveness	Growth and development
BnXTH1	MYC		Abiotic and biotic stress
BnXTH1	MYB recognition site		Abiotic and biotic stress
BnXTH1	ARE	cis-acting regulatory element essential for the anaerobic induction	Abiotic and biotic stress
BnXTH1	MYC		Abiotic and biotic stress
BnXTH1	Box 4	part of a conserved DNA module involved in light responsiveness	Growth and development
BnXTH1	G-Box	cis-acting regulatory element involved in light responsiveness	Growth and development
BnXTH1	MYC		Abiotic and biotic stress
BnXTH1	O_2-site	cis-acting regulatory element involved in zein metabolism regulation	Growth and development
BnXTH1	CGTCA-motif	cis-acting regulatory element involved in the MeJA-responsiveness	Growth and development
BnXTH1	ARE	cis-acting regulatory element essential for the anaerobic induction	Abiotic and biotic stress
BnXTH1	AT-rich element	binding site of AT-rich DNA binding protein (ATBP-1)	Growth and development
BnXTH1	ARE	cis-acting regulatory element essential for the anaerobic induction	Abiotic and biotic stress
BnXTH1	MYC		Abiotic and biotic stress
BnXTH1	TCT-motif	part of a light responsive element	Growth and development
BnXTH1	ERE		Phytohormione reponsive
BnXTH1	STRE		Abiotic and biotic stress
BnXTH2	Box 4	part of a conserved DNA module involved in light responsiveness	Growth and development

BnXTH2	O_2-site	cis-acting regulatory element involved in zein metabolism regulation	Growth and development
BnXTH2	MYB		Abiotic and biotic stress
BnXTH2	HD-Zip 3	protein binding site	Growth and development
BnXTH2	WUN-motif	wound-responsive element	Abiotic and biotic stress
BnXTH2	AAGAA-motif		Growth and development
BnXTH2	ARE	cis-acting regulatory element essential for the anaerobic induction	Abiotic and biotic stress
BnXTH2	AS-1		Abiotic and biotic stress
BnXTH2	TGACG-motif	cis-acting regulatory element involved in the MeJA-responsiveness	Phytohormione reponsive
BnXTH2	ABRE4		Phytohormione reponsive
BnXTH2	ARE	cis-acting regulatory element essential for the anaerobic induction	Abiotic and biotic stress
BnXTH2	MYB		Abiotic and biotic stress
BnXTH2	CGTCA-motif	cis-acting regulatory element involved in the MeJA-responsiveness	Growth and development
BnXTH2	TGA-element	auxin-responsive element	Phytohormione reponsive
BnXTH2	MYB		Abiotic and biotic stress
BnXTH3	ABRE	cis-acting element involved in the abscisic acid responsiveness	Phytohormione reponsive
BnXTH3	MYB		Abiotic and biotic stress
BnXTH3	WUN-motif	wound-responsive element	Abiotic and biotic stress
BnXTH3	STRE		Abiotic and biotic stress
BnXTH3	AT1-motif	part of a light responsive module	Growth and development
BnXTH3	Box 4	part of a conserved DNA module involved in light responsiveness	Growth and development
BnXTH3	STRE		Abiotic and biotic stress
BnXTH3	Sp1	light responsive element	Growth and development
BnXTH3	STRE		Abiotic and biotic stress
BnXTH3	MYC		Abiotic and biotic stress
BnXTH3	G-box	cis-acting regulatory element involved in light responsiveness	Growth and development
BnXTH3	ABRE3a		Phytohormione reponsive

BnXTH3	ABRE	cis-acting element involved in the abscisic acid responsiveness	Phytohormione reponsive
BnXTH3	Box 4	part of a conserved DNA module involved in light responsiveness	Growth and development
BnXTH3	MYC		Abiotic and biotic stress
BnXTH3	MYB		Abiotic and biotic stress
BnXTH3	ARE	cis-acting regulatory element essential for the anaerobic induction	Abiotic and biotic stress
BnXTH3	Myb-binding site		Abiotic and biotic stress
BnXTH3	MYB		Abiotic and biotic stress
BnXTH3	Myb-binding site		Abiotic and biotic stress
BnXTH3	MYB		Abiotic and biotic stress
BnXTH3	CGTCA-motif	cis-acting regulatory element involved in the MeJA-responsiveness	Growth and development
BnXTH3	MRE	MYB binding site involved in light responsiveness	Growth and development
BnXTH4	chs-CMA2a	part of a light responsive element	Growth and development
BnXTH4	MYB		Abiotic and biotic stress
BnXTH4	MYC		Abiotic and biotic stress
BnXTH4	Box 4	part of a conserved DNA module involved in light responsiveness	Growth and development
BnXTH4	ABRE4		Phytohormione reponsive
BnXTH4	WUN-motif	wound-responsive element	Abiotic and biotic stress
BnXTH4	Box 4	part of a conserved DNA module involved in light responsiveness	Growth and development
BnXTH4	Box 4	part of a conserved DNA module involved in light responsiveness	Growth and development
BnXTH4	Box 4	part of a conserved DNA module involved in light responsiveness	Growth and development
BnXTH4	Box 4	part of a conserved DNA module involved in light responsiveness	Growth and development
BnXTH4	MYC		Abiotic and biotic stress
BnXTH4	ACE	cis-acting element involved in light responsiveness	Growth and development
BnXTH4	AE-box	part of a module for light response	Growth and development
BnXTH4	MYB		Abiotic and biotic stress
BnXTH4	TC-rich repeats	cis-acting element involved in defense and stress responsiveness	Abiotic and biotic stress

BnXTH4	MYB recognition site		Abiotic and biotic stress
BnXTH4	G-box	cis-acting regulatory element involved in light responsiveness	Growth and development
BnXTH4	Box II	part of a light responsive element	Growth and development
BnXTH4	ABRE4		Phytohormione reponsive
BnXTH5	ARE	cis-acting regulatory element essential for the anaerobic induction	Abiotic and biotic stress
BnXTH5	W box		Abiotic and biotic stress
BnXTH5	AS-1		Abiotic and biotic stress
BnXTH5	TGACG-motif	cis-acting regulatory element involved in the MeJA-responsiveness	Phytohormione reponsive
BnXTH5	MYC		Abiotic and biotic stress
BnXTH5	ERE		Phytohormione reponsive
BnXTH5	ARE	cis-acting regulatory element essential for the anaerobic induction	Abiotic and biotic stress
BnXTH5	MYC		Abiotic and biotic stress
BnXTH5	Box 4	part of a conserved DNA module involved in light responsiveness	Growth and development
BnXTH5	Box 4	part of a conserved DNA module involved in light responsiveness	Growth and development
BnXTH5	ARE	cis-acting regulatory element essential for the anaerobic induction	Abiotic and biotic stress
BnXTH5	STRE		Abiotic and biotic stress
BnXTH5	AE-box	part of a module for light response	Growth and development
BnXTH5	AAGAA-motif		Growth and development
BnXTH5	MYB		Abiotic and biotic stress
BnXTH5	Myb-binding site		Abiotic and biotic stress
BnXTH5	ARE	cis-acting regulatory element essential for the anaerobic induction	Abiotic and biotic stress
BnXTH5	MYB		Abiotic and biotic stress
BnXTH5	MYB		Abiotic and biotic stress
BnXTH5	box S		Abiotic and biotic stress
BnXTH5	ARE	cis-acting regulatory element essential for the anaerobic induction	Abiotic and biotic stress
BnXTH5	CAT-box	cis-acting regulatory element related to meristem expression	Growth and development

BnXTH6	Box 4	part of a conserved DNA module involved in light responsiveness	Growth and development
BnXTH6	W box		Abiotic and biotic stress
BnXTH6	AT-rich element	binding site of AT-rich DNA binding protein (ATBP-1)	Growth and development
BnXTH6	MYC		Abiotic and biotic stress
BnXTH6	Box 4	part of a conserved DNA module involved in light responsiveness	Growth and development
BnXTH6	G-Box	cis-acting regulatory element involved in light responsiveness	Growth and development
BnXTH6	MYC		Abiotic and biotic stress
BnXTH6	ARE	cis-acting regulatory element essential for the anaerobic induction	Abiotic and biotic stress
BnXTH6	MYC		Abiotic and biotic stress
BnXTH6	Box 4	part of a conserved DNA module involved in light responsiveness	Growth and development
BnXTH6	GATA-motif	part of a light responsive element	Growth and development
BnXTH6	AS-1		Abiotic and biotic stress
BnXTH6	TGACG-motif	cis-acting regulatory element involved in the MeJA-responsiveness	Phytohormione reponsive
BnXTH6	Box 4	part of a conserved DNA module involved in light responsiveness	Growth and development
BnXTH6	Box 4	part of a conserved DNA module involved in light responsiveness	Growth and development
BnXTH6	Box 4	part of a conserved DNA module involved in light responsiveness	Growth and development
BnXTH6	ERE		Phytohormione reponsive
BnXTH6	ABRE3a		Phytohormione reponsive
BnXTH6	G-box	cis-acting regulatory element involved in light responsiveness	Growth and development
BnXTH6	ABRE	cis-acting element involved in the abscisic acid responsiveness	Phytohormione reponsive
BnXTH6	G-Box	cis-acting regulatory element involved in light responsiveness	Growth and development
BnXTH6	G-box	cis-acting regulatory element involved in light responsiveness	Growth and development
BnXTH6	ABRE4		Phytohormione reponsive
BnXTH6	ARE	cis-acting regulatory element essential for the anaerobic induction	Abiotic and biotic stress
BnXTH6	MBS	MYB binding site involved in drought-inducibility	Abiotic and biotic stress
BnXTH6	MYB		Abiotic and biotic stress

BnXTH6	TCA-element	cis-acting element involved in salicylic acid responsiveness	Phytohormione reponsive
BnXTH6	STRE		Abiotic and biotic stress
BnXTH7	Box 4	part of a conserved DNA module involved in light responsiveness	Growth and development
BnXTH7	CGTCA-motif	cis-acting regulatory element involved in the MeJA-responsiveness	Growth and development
BnXTH7	circadian	cis-acting regulatory element involved in circadian control	Growth and development
BnXTH7	ARE	cis-acting regulatory element essential for the anaerobic induction	Abiotic and biotic stress
BnXTH7	DRE core		Abiotic and biotic stress
BnXTH7	MYC		Abiotic and biotic stress
BnXTH7	G-box	cis-acting regulatory element involved in light responsiveness	Growth and development
BnXTH7	TGACG-motif	cis-acting regulatory element involved in the MeJA-responsiveness	Phytohormione reponsive
BnXTH7	AS-1		Abiotic and biotic stress
BnXTH7	ABRE	cis-acting element involved in the abscisic acid responsiveness	Phytohormione reponsive
BnXTH7	TCT-motif	part of a light responsive element	Growth and development
BnXTH7	ABRE	cis-acting element involved in the abscisic acid responsiveness	Phytohormione reponsive
BnXTH7	G-box	cis-acting regulatory element involved in light responsiveness	Growth and development
BnXTH7	ABRE	cis-acting element involved in the abscisic acid responsiveness	Phytohormione reponsive
BnXTH7	ABRE	cis-acting element involved in the abscisic acid responsiveness	Phytohormione reponsive
BnXTH7	ABRE	cis-acting element involved in the abscisic acid responsiveness	Phytohormione reponsive
BnXTH7	TGACG-motif	cis-acting regulatory element involved in the MeJA-responsiveness	Phytohormione reponsive
BnXTH7	AS-1		Abiotic and biotic stress
BnXTH7	ABRE	cis-acting element involved in the abscisic acid responsiveness	Phytohormione reponsive
BnXTH8	GT1-motif	light responsive element	Growth and development
BnXTH8	WRE3		Abiotic and biotic stress
BnXTH8	ABRE	cis-acting element involved in the abscisic acid responsiveness	Phytohormione reponsive
BnXTH8	CAT-box	cis-acting regulatory element related to meristem expression	Growth and development
BnXTH8	ABRE	cis-acting element involved in the abscisic acid responsiveness	Phytohormione reponsive

BnXTH8	G-box	cis-acting regulatory element involved in light responsiveness	Growth and development
BnXTH8	ABRE2		Phytohormione reponsive
BnXTH8	Box II	part of a light responsive element	Growth and development
BnXTH8	ABRE	cis-acting element involved in the abscisic acid responsiveness	Phytohormione reponsive
BnXTH8	G-box	cis-acting regulatory element involved in light responsiveness	Growth and development
BnXTH8	G-Box	cis-acting regulatory element involved in light responsiveness	Growth and development
BnXTH8	ABRE	cis-acting element involved in the abscisic acid responsiveness	Phytohormione reponsive
BnXTH8	MYC		Abiotic and biotic stress
BnXTH8	G-box	cis-acting regulatory element involved in light responsiveness	Growth and development
BnXTH8	ABRE2		Phytohormione reponsive
BnXTH8	Box II	part of a light responsive element	Growth and development
BnXTH8	ABRE	cis-acting element involved in the abscisic acid responsiveness	Phytohormione reponsive
BnXTH8	G-box	cis-acting regulatory element involved in light responsiveness	Growth and development
BnXTH8	G-Box	cis-acting regulatory element involved in light responsiveness	Growth and development
BnXTH8	ABRE	cis-acting element involved in the abscisic acid responsiveness	Phytohormione reponsive
BnXTH8	G-Box	cis-acting regulatory element involved in light responsiveness	Growth and development
BnXTH8	WRE3		Abiotic and biotic stress
BnXTH8	ATC-motif	part of a conserved DNA module involved in light responsiveness	Growth and development
BnXTH8	MYC		Abiotic and biotic stress
BnXTH8	Box II	part of a light responsive element	Growth and development
BnXTH8	ABRE	cis-acting element involved in the abscisic acid responsiveness	Phytohormione reponsive
BnXTH8	ABRE3a		Phytohormione reponsive
BnXTH8	G-box	cis-acting regulatory element involved in light responsiveness	Growth and development
BnXTH8	ABRE	cis-acting element involved in the abscisic acid responsiveness	Phytohormione reponsive
BnXTH8	MRE	MYB binding site involved in light responsiveness	Growth and development
BnXTH8	MBS	MYB binding site involved in drought-inducibility	Abiotic and biotic stress

BnXTH8	MYB		Abiotic and biotic stress
BnXTH8	MYB		Abiotic and biotic stress
BnXTH8	ABRE	cis-acting element involved in the abscisic acid responsiveness	Phytohormione reponsive
BnXTH8	P-box	gibberellin-responsive element	Phytohormione reponsive
BnXTH8	TCA-element	cis-acting element involved in salicylic acid responsiveness	Phytohormione reponsive
BnXTH9	TGA-element	auxin-responsive element	Phytohormione reponsive
BnXTH9	TGA-element	auxin-responsive element	Phytohormione reponsive
BnXTH9	ERE		Phytohormione reponsive
BnXTH9	TGA-element	auxin-responsive element	Phytohormione reponsive
BnXTH9	ARE	cis-acting regulatory element essential for the anaerobic induction	Abiotic and biotic stress
BnXTH9	TGA-element	auxin-responsive element	Phytohormione reponsive
BnXTH9	TGA-element	auxin-responsive element	Phytohormione reponsive
BnXTH9	TGA-element	auxin-responsive element	Phytohormione reponsive
BnXTH9	TGA-element	auxin-responsive element	Phytohormione reponsive
BnXTH9	AE-box	part of a module for light response	Growth and development
BnXTH9	TGA-element	auxin-responsive element	Phytohormione reponsive
BnXTH9	TGA-element	auxin-responsive element	Phytohormione reponsive
BnXTH9	MBS	MYB binding site involved in drought-inducibility	Abiotic and biotic stress
BnXTH9	MYB		Abiotic and biotic stress
BnXTH9	TGA-element	auxin-responsive element	Phytohormione reponsive
BnXTH9	TGA-element	auxin-responsive element	Phytohormione reponsive
BnXTH9	TGA-element	auxin-responsive element	Phytohormione reponsive
BnXTH9	GA-motif	part of a light responsive element	Growth and development
BnXTH9	MRE	MYB binding site involved in light responsiveness	Growth and development
BnXTH9	TGA-element	auxin-responsive element	Phytohormione reponsive
BnXTH9	AE-box	part of a module for light response	Growth and development

BnXTH9	ARE	cis-acting regulatory element essential for the anaerobic induction	Abiotic and biotic stress
BnXTH9	TGA-element	auxin-responsive element	Phytohormione reponsive
BnXTH9	TGA-element	auxin-responsive element	Phytohormione reponsive
BnXTH9	TGA-element	auxin-responsive element	Phytohormione reponsive
BnXTH9	TGA-element	auxin-responsive element	Phytohormione reponsive
BnXTH9	LTR	cis-acting element involved in low-temperature responsiveness	Abiotic and biotic stress
BnXTH9	TGA-element	auxin-responsive element	Phytohormione reponsive
BnXTH9	I-box	part of a light responsive element	Growth and development
BnXTH9	I-box	part of a light responsive element	Growth and development
BnXTH9	W box		Abiotic and biotic stress
BnXTH9	CAT-box	cis-acting regulatory element related to meristem expression	Growth and development
BnXTH9	CCAAT-box	MYBHv1 binding site	Growth and development
BnXTH9	STRE		Abiotic and biotic stress
BnXTH10	TGACG-motif	cis-acting regulatory element involved in the MeJA-responsiveness	Phytohormione reponsive
BnXTH10	AS-1		Abiotic and biotic stress
BnXTH10	STRE		Abiotic and biotic stress
BnXTH10	Myb-binding site		Abiotic and biotic stress
BnXTH10	MYB		Abiotic and biotic stress
BnXTH10	Box 4	part of a conserved DNA module involved in light responsiveness	Growth and development
BnXTH10	Myb-binding site		Abiotic and biotic stress
BnXTH10	MYB		Abiotic and biotic stress
BnXTH10	MYB		Abiotic and biotic stress
BnXTH10	WRE3		Abiotic and biotic stress
BnXTH10	CGTCA-motif	cis-acting regulatory element involved in the MeJA-responsiveness	Growth and development
BnXTH10	MYC		Abiotic and biotic stress
BnXTH10	STRE		Abiotic and biotic stress

BnXTH10	TCT-motif	part of a light responsive element	Growth and development
BnXTH10	STRE		Abiotic and biotic stress
BnXTH10	CCAAT-box	MYBHv1 binding site	Growth and development
BnXTH10	CAT-box	cis-acting regulatory element related to meristem expression	Growth and development
BnXTH10	MBS	MYB binding site involved in drought-inducibility	Abiotic and biotic stress
BnXTH10	MYB		Abiotic and biotic stress
BnXTH10	GATA-motif	part of a light responsive element	Growth and development
BnXTH10	ABRE	cis-acting element involved in the abscisic acid responsiveness	Phytohormione reponsive
BnXTH10	MYB		Abiotic and biotic stress
BnXTH10	ABRE	cis-acting element involved in the abscisic acid responsiveness	Phytohormione reponsive
BnXTH10	MYC		Abiotic and biotic stress
BnXTH10	TCT-motif	part of a light responsive element	Growth and development
BnXTH10	WRE3		Abiotic and biotic stress
BnXTH10	Myb-binding site		Abiotic and biotic stress
BnXTH10	MYB		Abiotic and biotic stress
BnXTH10	ARE	cis-acting regulatory element essential for the anaerobic induction	Abiotic and biotic stress
BnXTH10	WUN-motif	wound-responsive element	Abiotic and biotic stress
BnXTH10	LTR	cis-acting element involved in low-temperature responsiveness	Abiotic and biotic stress
BnXTH11	TCT-motif	part of a light responsive element	Growth and development
BnXTH11	TCT-motif	part of a light responsive element	Growth and development
BnXTH11	AAGAA-motif		Growth and development
BnXTH11	AS-1		Abiotic and biotic stress
BnXTH11	TGACG-motif	cis-acting regulatory element involved in the MeJA-responsiveness	Phytohormione reponsive
BnXTH11	ABRE	cis-acting element involved in the abscisic acid responsiveness	Phytohormione reponsive
BnXTH11	ABRE3a		Phytohormione reponsive
BnXTH11	G-box	cis-acting regulatory element involved in light responsiveness	Growth and development

BnXTH11	ABRE	cis-acting element involved in the abscisic acid responsiveness	Phytohormione reponsive
BnXTH11	ERE		Phytohormione reponsive
BnXTH11	MYC		Abiotic and biotic stress
BnXTH11	AAGAA-motif		Growth and development
BnXTH11	AE-box	part of a module for light response	Growth and development
BnXTH11	ARE	cis-acting regulatory element essential for the anaerobic induction	Abiotic and biotic stress
BnXTH11	MYB		Abiotic and biotic stress
BnXTH11	circadian	cis-acting regulatory element involved in circadian control	Growth and development
BnXTH12	box S		Abiotic and biotic stress
BnXTH12	WRE3		Abiotic and biotic stress
BnXTH12	TGACG-motif	cis-acting regulatory element involved in the MeJA-responsiveness	Phytohormione reponsive
BnXTH12	AS-1		Abiotic and biotic stress
BnXTH12	chs-CMA2a	part of a light responsive element	Growth and development
BnXTH12	O_2-site	cis-acting regulatory element involved in zein metabolism regulation	Growth and development
BnXTH12	LTR	cis-acting element involved in low-temperature responsiveness	Abiotic and biotic stress
BnXTH12	Box 4	part of a conserved DNA module involved in light responsiveness	Growth and development
BnXTH12	G-box	cis-acting regulatory element involved in light responsiveness	Growth and development
BnXTH12	TGACG-motif	cis-acting regulatory element involved in the MeJA-responsiveness	Phytohormione reponsive
BnXTH12	AS-1		Abiotic and biotic stress
BnXTH12	AE-box	part of a module for light response	Growth and development
BnXTH12	MYC		Abiotic and biotic stress
BnXTH12	TCT-motif	part of a light responsive element	Growth and development
BnXTH12	O_2-site	cis-acting regulatory element involved in zein metabolism regulation	Growth and development
BnXTH12	MYC		Abiotic and biotic stress
BnXTH12	CAT-box	cis-acting regulatory element related to meristem expression	Growth and development
BnXTH12	G-box	cis-acting regulatory element involved in light responsiveness	Growth and development

BnXTH12	MYC		Abiotic and biotic stress
BnXTH12	W box		Abiotic and biotic stress
BnXTH12	TGA-element	auxin-responsive element	Phytohormione reponsive
BnXTH13	P-box	gibberellin-responsive element	Phytohormione reponsive
BnXTH13	MYB		Abiotic and biotic stress
BnXTH13	GARE-motif	gibberellin-responsive element	Growth and development
BnXTH13	GT1-motif	light responsive element	Growth and development
BnXTH13	MYC		Abiotic and biotic stress
BnXTH13	GATA-motif	part of a light responsive element	Growth and development
BnXTH13	GARE-motif	gibberellin-responsive element	Growth and development
BnXTH13	ATC-motif	part of a conserved DNA module involved in light responsiveness	Growth and development
BnXTH13	AAGAA-motif		Growth and development
BnXTH13	AE-box	part of a module for light response	Growth and development
BnXTH13	MYB		Abiotic and biotic stress
BnXTH13	MYB		Abiotic and biotic stress
BnXTH13	G-box	cis-acting regulatory element involved in light responsiveness	Growth and development
BnXTH13	ARE	cis-acting regulatory element essential for the anaerobic induction	Abiotic and biotic stress
BnXTH13	F-box		Growth and development
BnXTH13	G-box	cis-acting regulatory element involved in light responsiveness	Growth and development
BnXTH13	CGTCA-motif	cis-acting regulatory element involved in the MeJA-responsiveness	Growth and development
BnXTH13	MYB		Abiotic and biotic stress
BnXTH13	CCGTCC-box		Growth and development
BnXTH13	I-box	part of a light responsive element	Growth and development
BnXTH13	ABRE	cis-acting element involved in the abscisic acid responsiveness	Phytohormione reponsive
BnXTH13	ABRE3a		Phytohormione reponsive
BnXTH13	G-box	cis-acting regulatory element involved in light responsiveness	Growth and development

BnXTH13	ABRE	cis-acting element involved in the abscisic acid responsiveness	Phytohormione reponsive
BnXTH13	TCT-motif	part of a light responsive element	Growth and development
BnXTH13	STRE		Abiotic and biotic stress
BnXTH13	MYB		Abiotic and biotic stress
BnXTH14	CAT-box	cis-acting regulatory element related to meristem expression	Growth and development
BnXTH14	CGTCA-motif	cis-acting regulatory element involved in the MeJA-responsiveness	Growth and development
BnXTH14	G-box	cis-acting regulatory element involved in light responsiveness	Growth and development
BnXTH14	I-box	part of a light responsive element	Growth and development
BnXTH14	MYB		Abiotic and biotic stress
BnXTH14	MYB		Abiotic and biotic stress
BnXTH14	MYB		Abiotic and biotic stress
BnXTH14	Myb-binding site		Abiotic and biotic stress
BnXTH14	MYC		Abiotic and biotic stress
BnXTH14	MYC		Abiotic and biotic stress
BnXTH14	Sp1	light responsive element	Growth and development
BnXTH14	STRE		Abiotic and biotic stress
BnXTH14	TCT-motif	part of a light responsive element	Growth and development
BnXTH14	TCT-motif	part of a light responsive element	Growth and development
BnXTH15	MYB recognition site		Abiotic and biotic stress
BnXTH15	ERE		Phytohormione reponsive
BnXTH15	ERE		Phytohormione reponsive
BnXTH15	ERE		Phytohormione reponsive
BnXTH15	G-box	cis-acting regulatory element involved in light responsiveness	Growth and development
BnXTH15	ABRE4		Phytohormione reponsive
BnXTH15	GT1-motif	light responsive element	Growth and development
BnXTH15	MYC		Abiotic and biotic stress

BnXTH15	ERE		Phytohormione reponsive
BnXTH15	G-box	cis-acting regulatory element involved in light responsiveness	Growth and development
BnXTH15	G-Box	cis-acting regulatory element involved in light responsiveness	Growth and development
BnXTH15	ABRE	cis-acting element involved in the abscisic acid responsiveness	Phytohormione reponsive
BnXTH15	G-box	cis-acting regulatory element involved in light responsiveness	Growth and development
BnXTH15	ABRE	cis-acting element involved in the abscisic acid responsiveness	Phytohormione reponsive
BnXTH15	MYB		Abiotic and biotic stress
BnXTH15	ABRE4		Phytohormione reponsive
BnXTH15	ERE		Phytohormione reponsive
BnXTH15	ARE	cis-acting regulatory element essential for the anaerobic induction	Abiotic and biotic stress
BnXTH15	ARE	cis-acting regulatory element essential for the anaerobic induction	Abiotic and biotic stress
BnXTH16	Box 4	part of a conserved DNA module involved in light responsiveness	Growth and development
BnXTH16	Box 4	part of a conserved DNA module involved in light responsiveness	Growth and development
BnXTH16	Box 4	part of a conserved DNA module involved in light responsiveness	Growth and development
BnXTH16	MYB		Abiotic and biotic stress
BnXTH16	MYC		Abiotic and biotic stress
BnXTH16	TC-rich repeats	cis-acting element involved in defense and stress responsiveness	Abiotic and biotic stress
BnXTH16	Box 4	part of a conserved DNA module involved in light responsiveness	Growth and development
BnXTH16	MBSI	MYB binding site involved in flavonoid biosynthetic genes regulation	Abiotic and biotic stress
BnXTH16	RY-element	cis-acting regulatory element involved in seed-specific regulation	Phytohormione reponsive
BnXTH16	GT1-motif	light responsive element	Growth and development
BnXTH16	GT1-motif	light responsive element	Growth and development
BnXTH16	ABRE	cis-acting element involved in the abscisic acid responsiveness	Phytohormione reponsive
BnXTH16	ABRE2		Phytohormione reponsive
BnXTH16	Box II	part of a light responsive element	Growth and development
BnXTH16	ABRE	cis-acting element involved in the abscisic acid responsiveness	Phytohormione reponsive

BnXTH16	G-Box	cis-acting regulatory element involved in light responsiveness	Growth and development
BnXTH16	G-box	cis-acting regulatory element involved in light responsiveness	Growth and development
BnXTH16	ABRE	cis-acting element involved in the abscisic acid responsiveness	Phytohormione reponsive
BnXTH16	MYB		Abiotic and biotic stress
BnXTH16	W box		Abiotic and biotic stress
BnXTH16	GC-motif	enhancer-like element involved in anoxic specific inducibility	Abiotic and biotic stress
BnXTH16	MYC		Abiotic and biotic stress
BnXTH16	MYC		Abiotic and biotic stress
BnXTH16	CGTCA-motif	cis-acting regulatory element involved in the MeJA-responsiveness	Growth and development
BnXTH16	MYC		Abiotic and biotic stress
BnXTH16	TC-rich repeats	cis-acting element involved in defense and stress responsiveness	Abiotic and biotic stress
BnXTH16	MYC		Abiotic and biotic stress
BnXTH16	MYC		Abiotic and biotic stress
BnXTH16	chs-CMA2a	part of a light responsive element	Growth and development
BnXTH16	MBSI	MYB binding site involved in flavonoid biosynthetic genes regulation	Abiotic and biotic stress
BnXTH16	GT1-motif	light responsive element	Growth and development
BnXTH16	MYC		Abiotic and biotic stress
BnXTH16	Myc		Abiotic and biotic stress
BnXTH16	W box		Abiotic and biotic stress
BnXTH16	MYB		Abiotic and biotic stress
BnXTH17	MYB		Abiotic and biotic stress
BnXTH17	Box 4	part of a conserved DNA module involved in light responsiveness	Growth and development
BnXTH17	CGTCA-motif	cis-acting regulatory element involved in the MeJA-responsiveness	Growth and development
BnXTH17	GA-motif	part of a light responsive element	Growth and development
BnXTH17	MYB		Abiotic and biotic stress
BnXTH17	Box 4	part of a conserved DNA module involved in light responsiveness	Growth and development

BnXTH17	AS-1		Abiotic and biotic stress
BnXTH17	TGACG-motif	cis-acting regulatory element involved in the MeJA-responsiveness	Phytohormione reponsive
BnXTH17	MYC		Abiotic and biotic stress
BnXTH17	G-box	cis-acting regulatory element involved in light responsiveness	Growth and development
BnXTH17	ABRE	cis-acting element involved in the abscisic acid responsiveness	Phytohormione reponsive
BnXTH17	ABRE3a		Phytohormione reponsive
BnXTH17	G-box	cis-acting regulatory element involved in light responsiveness	Growth and development
BnXTH17	ABRE	cis-acting element involved in the abscisic acid responsiveness	Phytohormione reponsive
BnXTH17	Sp1	light responsive element	Growth and development
BnXTH17	G-Box	cis-acting regulatory element involved in light responsiveness	Growth and development
BnXTH17	ACE	cis-acting element involved in light responsiveness	Growth and development
BnXTH17	GT1-motif	light responsive element	Growth and development
BnXTH17	G-Box	cis-acting regulatory element involved in light responsiveness	Growth and development
BnXTH17	ERE		Phytohormione reponsive
BnXTH17	AS-1		Abiotic and biotic stress
BnXTH17	TGACG-motif	cis-acting regulatory element involved in the MeJA-responsiveness	Phytohormione reponsive
BnXTH17	MYB		Abiotic and biotic stress
BnXTH17	ATCT-motif	part of a conserved DNA module involved in light responsiveness	Growth and development
BnXTH17	MRE	MYB binding site involved in light responsiveness	Growth and development
BnXTH17	MYB		Abiotic and biotic stress
BnXTH17	TCA-element	cis-acting element involved in salicylic acid responsiveness	Phytohormione reponsive
BnXTH18	Box 4	part of a conserved DNA module involved in light responsiveness	Growth and development
BnXTH18	ERE		Phytohormione reponsive
BnXTH18	3-AF1 binding site	light responsive element	Growth and development
BnXTH18	TCCC-motif	part of a light responsive element	Growth and development
BnXTH18	TCCC-motif	part of a light responsive element	Growth and development

BnXTH18	MYB		Abiotic and biotic stress
BnXTH18	WRE3		Abiotic and biotic stress
BnXTH18	TCCC-motif	part of a light responsive element	Growth and development
BnXTH18	WRE3		Abiotic and biotic stress
BnXTH18	WUN-motif	wound-responsive element	Abiotic and biotic stress
BnXTH18	chs-Unit 1 m1	part of a light responsive element	Growth and development
BnXTH18	MYB		Abiotic and biotic stress
BnXTH18	TCA-element	cis-acting element involved in salicylic acid responsiveness	Phytohormione reponsive
BnXTH18	ERE		Phytohormione reponsive
BnXTH18	circadian	cis-acting regulatory element involved in circadian control	Growth and development
BnXTH18	TCT-motif	part of a light responsive element	Growth and development
BnXTH18	MYC		Abiotic and biotic stress
BnXTH18	G-box	cis-acting regulatory element involved in light responsiveness	Growth and development
BnXTH18	ARE	cis-acting regulatory element essential for the anaerobic induction	Abiotic and biotic stress
BnXTH18	MYC		Abiotic and biotic stress
BnXTH18	WUN-motif	wound-responsive element	Abiotic and biotic stress
BnXTH18	I-box	part of a light responsive element	Growth and development
BnXTH18	TC-rich repeats	cis-acting element involved in defense and stress responsiveness	Abiotic and biotic stress
BnXTH18	AS-1		Abiotic and biotic stress
BnXTH18	TGACG-motif	cis-acting regulatory element involved in the MeJA-responsiveness	Phytohormione reponsive
BnXTH18	TCCC-motif	part of a light responsive element	Growth and development
BnXTH18	MBS	MYB binding site involved in drought-inducibility	Abiotic and biotic stress
BnXTH18	MYB		Abiotic and biotic stress
BnXTH18	MYB		Abiotic and biotic stress
BnXTH18	Myb-binding site		Abiotic and biotic stress
BnXTH18	box S		Abiotic and biotic stress

BnXTH18	G-Box	cis-acting regulatory element involved in light responsiveness	Growth and development
BnXTH18	ARE	cis-acting regulatory element essential for the anaerobic induction	Abiotic and biotic stress
BnXTH19	ERE		Phytohormione reponsive
BnXTH19	ERE		Phytohormione reponsive
BnXTH19	ERE		Phytohormione reponsive
BnXTH19	ARE	cis-acting regulatory element essential for the anaerobic induction	Abiotic and biotic stress
BnXTH19	ARE	cis-acting regulatory element essential for the anaerobic induction	Abiotic and biotic stress
BnXTH19	ARE	cis-acting regulatory element essential for the anaerobic induction	Abiotic and biotic stress
BnXTH19	ARE	cis-acting regulatory element essential for the anaerobic induction	Abiotic and biotic stress
BnXTH19	CCAAT-box	MYBHv1 binding site	Growth and development
BnXTH19	AAGAA-motif		Growth and development
BnXTH19	AAGAA-motif		Growth and development
BnXTH19	P-box	gibberellin-responsive element	Phytohormione reponsive
BnXTH19	ABRE3a		Phytohormione reponsive
BnXTH19	TC-rich repeats	cis-acting element involved in defense and stress responsiveness	Abiotic and biotic stress
BnXTH19	WRE3		Abiotic and biotic stress
BnXTH19	WRE3		Abiotic and biotic stress
BnXTH19	GT1-motif	light responsive element	Growth and development
BnXTH19	GT1-motif	light responsive element	Growth and development
BnXTH19	CGTCA-motif	cis-acting regulatory element involved in the MeJA-responsiveness	Growth and development
BnXTH19	CGTCA-motif	cis-acting regulatory element involved in the MeJA-responsiveness	Growth and development
BnXTH19	STRE		Abiotic and biotic stress
BnXTH19	ABRE	cis-acting element involved in the abscisic acid responsiveness	Phytohormione reponsive
BnXTH19	ABRE	cis-acting element involved in the abscisic acid responsiveness	Phytohormione reponsive
BnXTH19	TGA-element	auxin-responsive element	Phytohormione reponsive
BnXTH19	MYB		Abiotic and biotic stress

BnXTH19	G-box	cis-acting regulatory element involved in light responsiveness	Growth and development
BnXTH19	G-box	cis-acting regulatory element involved in light responsiveness	Growth and development
BnXTH19	G-box	cis-acting regulatory element involved in light responsiveness	Growth and development
BnXTH19	WUN-motif	wound-responsive element	Abiotic and biotic stress
BnXTH19	WUN-motif	wound-responsive element	Abiotic and biotic stress
BnXTH19	WUN-motif	wound-responsive element	Abiotic and biotic stress

Table S4. Primers used in this study

Primer name	Squences (5' to 3')	Usage
BnXTH1_qF	AGCATATGGAACGCCGACGATTG	qRT-PCR
BnXTH1_qR	GAGAGGTGCCAAGCCTTGTACTG	qRT-PCR
BnXTH2_qF	CGTACACGGTGCAGACTAACATCTTC	qRT-PCR
BnXTH2_qR	GTAAGTGTGGTAAGCCGCCGAAG	qRT-PCR
BnXTH3_qF	CCTATGGAACGCCGACGATTGG	qRT-PCR
BnXTH3_qR	CGCCGTTGAGGTCTTGGAACTC	qRT-PCR
BnXTH4_qF	GCTGTGTTGTTATTGTCTGGTCTTGTC	qRT-PCR
BnXTH4_qR	GAGGTTGCTGTCGCCGAAGAG	qRT-PCR
BnXTH5_qF	TCTCTTCATTGTCCTCTCAGTCATGTG	qRT-PCR
BnXTH5_qR	GAGTTGCTATTGATCCATTGCCAGTG	qRT-PCR
BnXTH6_qF	CTGGAACCCAACAGAAATCA	qRT-PCR
BnXTH6_qR	GTCCCATATCGACCCGTAA	qRT-PCR
BnXTH7_qF	CTGCTGGAACTGTCACTGCTT	qRT-PCR
BnXTH7_qR	TTGCCAACACCATTCACGT	qRT-PCR
BnXTH8_qF	GGTGGACAACATTCCGATTA	qRT-PCR
BnXTH8_qR	CGGCGTTGAAGTTCCTGTA	qRT-PCR
BnXTH9_qF	AGCGGACAACATACCAATAAGA	qRT-PCR
BnXTH9_qR	CGGTAGAATGCGGTGAAGG	qRT-PCR
BnXTH10_qF	CCCACCGCAGGATTACATT	qRT-PCR
BnXTH10_qR	CCTTGCTCCAGTTGATCTTCTC	qRT-PCR
BnXTH11_qF	TTTGCTGATGGGTTGGATA	qRT-PCR
BnXTH11_qR	CCTTGTCTGCGTGGTTTCT	qRT-PCR
BnXTH12_qF	TGGTGGAATGACGAGGAGC	qRT-PCR
BnXTH12_qR	TGCGAGGTTAGGTAGAAAGCAGT	qRT-PCR
BnXTH13_qF	CTCAGGGTCTGCTGTTCAATC	qRT-PCR
BnXTH13_qR	CGAAATCTATCTCGTCGTGTTT	qRT-PCR
BnXTH14_qF	GGCAAGACGAAAGCAACCA	qRT-PCR
BnXTH14_qR	CTGAGAAACAAATCCAGACCCT	qRT-PCR
BnXTH15_qF	CCAAGAACCAGCCAATGAG	qRT-PCR
BnXTH15_qR	TTGGTCTGCCAAGTGGAGT	qRT-PCR
BnXTH16_qF	GGGAAAGTCATTGGCAGAG	qRT-PCR
BnXTH16_qR	TGGGCACATCATCAACAAG	qRT-PCR
BnXTH17_qF	CGAGCGAAGATACTGAACG	qRT-PCR
BnXTH17_qR	AGAGTCTCCAGGGACAAGC	qRT-PCR
BnXTH18_qF	GGAACCGTCACTGCCTATT	qRT-PCR
BnXTH18_qR	GCCTTTGCCTTGACTGAAC	qRT-PCR
BnXTH19_qF	AAAGAGCCAGCCAATGAGG	qRT-PCR
BnXTH19_qR	GGGCGTCGAAGTTGCGATA	qRT-PCR
BnActin_qF	CGTTGAACCCTAAGGC	qRT-PCR
BnActin_qR	ATCCAGCACGATACCAG	qRT-PCR
p426-BnXTH1-F	AGAACTAGTGGATCCCCCGGGATGGGAGGAACAACAGTCCCT	Expression in yeast

p426-BnXTH1-R	ATTACATGACTCGAGGTCGACCTCTGGCAAAGTCGGGAAG	Expression in yeast
p426-BnXTH3-F	AGAACTAGTGGATCCCCCGGGATGGCTTGTCACTTGCAATGG	Expression in yeast
p426-BnXTH3-R	ATTACATGACTCGAGGTCGACTCACACATCGCGGTCCCTCTT	Expression in yeast
p426-BnXTH6-F	AGAACTAGTGGATCCCCCGGGTTGGAAAAGCTTGATCATCTC	Expression in yeast
p426-BnXTH6-R	ATTACATGACTCGAGGTCGACTTAGAATCTGGACTTCTTGCAC	Expression in yeast
p426-BnXTH15-F	AGAACTAGTGGATCCCCCGGGATGCCTTCCAAAACCTCTCTCC	Expression in yeast
p426-BnXTH15-R	ATTACATGACTCGAGGTCGACTTAGCACTCGGGTGTGTGTGTAT	Expression in yeast
